# Sleep characteristics and dementia: a multivariate Mendelian randomization study

**DOI:** 10.1002/alz70860_102935

**Published:** 2025-12-23

**Authors:** Yunyun Guo, Rui Wang, Shireen Sindi, Lefkos T Middleton, Miia Kivipelto

**Affiliations:** ^1^ The Ageing Epidemiology (AGE) Research Unit, School of Public Health, Imperial College London, London, United Kingdom; ^2^ Karolinska Institutet, Department of Neurobiology, Care Sciences and Society, Division of Clinical Geriatrics, Center for Alzheimer Research, Stockholm, Sweden; ^3^ Department of Physical Activity and Health, the Swedish School of Sport and Health Sciences, Stockholm, Sweden; ^4^ Wisconsin Alzheimer's Disease Research Center, School of Medicine and Public Health, University of Wisconsin, Madison, WI, USA; ^5^ Department of Neurology, Institute of Clinical Medicine, University of Eastern Finland, Kuopio, Finland; ^6^ Theme Inflammation and Aging, Karolinska University Hospital, Stockholm, Sweden; ^7^ Department of Neurobiology, Care Sciences and Society, Centre for Alzheimer Research, Division of Clinical Geriatrics, Karolinska Institutet, Stockholm, Sweden; ^8^ Institute of Public Health and Clinical Nutrition, University of Eastern Finland, Kuopio, Finland

## Abstract

**Background:**

Observational studies have provided inconsistent association between sleep characteristics and dementia, and the causal nature of this relationship remains unclear. This study aimed to investigate the comprehensive relationship between sleep characteristics and dementia using a bidirectional and multivariate Mendelian randomization (MR).

**Method:**

Summarized single nucleotide polymorphism (SNP) data involved in this study was from genome‐wide association studies, including risk genes of multiple sleep characteristics (i.e., daytime napping, daytime sleepiness, sleep duration, insomnia, and being a morning person) and all‐cause and cause‐specific dementia (e.g., Alzheimer's disease [AD], Lewy body dementia, and vascular dementia [VaD]). Independent genetic variants associated with sleep characteristics and dementia were used as instrumental variables. Inverse‐variance weighted (IVW) test, supplemented by MR‐Egger and weighted median estimators, was used as the main analyses. Multivariate MR employed the multivariate IVW test to adjust for potential confounders, including education, body mass index, smoking, alcohol consumption, hypertension, low‐density lipoprotein (LDL) cholesterol, hearing loss, physical activity, depression, traumatic brain injury, and diabetes.

**Result:**

When genetic variates of sleep characteristics were introduced as instrumental variables, a significant causal relationship between insomnia and AD (OR_IVW_ = 3.02, 95% CI: 1.28—7.10, *p* = 0.01) was observed in the univariate MR. But this relationship was largely diluted after adjusting for education or LDL cholesterol in the Multivariate MR. When genetic variants of dementia were treated as instrumental variables in IVW analyses, all cause dementia was associated with the characteristic of being a morning person, with significant ORs ranging from 1.003 to 1.005 (*p* < 0.05). Instrumental variables of VaD and Lewy body dementia were associated with shorter sleep durations (*p* < 0.05).

**Conclusion:**

The effect of insomnia on AD may largely modulated by education level and mediated by LDL cholesterol level. Future investigations are required to further verify the reversed relationship from dementia to other sleep characteristics, e.g., a shorter duration.

